# Pseudomyopia: A Review

**DOI:** 10.3390/vision6010017

**Published:** 2022-03-04

**Authors:** María García-Montero, Gema Felipe-Márquez, Pedro Arriola-Villalobos, Nuria Garzón

**Affiliations:** 1 Optometry and Vision Department, Faculty of Optics and Optometry, Complutense University of Madrid, 28037 Madrid, Spain; mgarzonj@ucm.es; 2 Servicio de Oftalmología, Hospital Clínico San Carlos, 28040 Madrid, Spain; gemafelipe@gmail.com (G.F.-M.); pedroarr@ucm.es (P.A.-V.)

**Keywords:** pseudomyopia, refractive error, accommodation, accommodation spasm

## Abstract

This review has identified evidence about pseudomyopia as the result of an increase in ocular refractive power due to an overstimulation of the eye’s accommodative mechanism. It cannot be confused with the term “secondary myopia”, which includes transient myopic shifts caused by lenticular refractive index changes and myopia associated with systemic syndromes. The aim was to synthesize the literature on qualitative evidence about pseudomyopia in terms that clarify its pathophysiology, clinical presentation, assessment and diagnosis and treatment. A comprehensive literature search of PubMed and the Scopus database was carried out for articles published up to November 2021, without a data limit. This review was reported following the preferred reporting items for systematic reviews and meta-analyses (PRISMA) guidelines. Following inclusion and exclusion criteria, a total of 54 studies were included in the qualitative synthesis. The terms pseudomyopia and accommodation spasm have been found in most of the studies reviewed. The review has warned that although there is agreement on the assessment and diagnosis of the condition, there is no consensus on its management, and the literature describes a range of treatment.

## 1. Introduction

The qualitative definition of the term “myopia” suggested by the International Myopia Institute (IMI) is “a refractive error in which rays of light entering the eye parallel to the optic axis are brought to a focus in front of the retina when ocular accommodation is relaxed. This usually results from the eyeball being too long from front to back, but can be caused by an overly curved cornea and/or a lens with increased optical power…” [1]. Considering the term “pseudo” from the Greek pseudes, meaning false or erroneous, there are some transient forms of myopia that are often termed “pseudomyopia”. However, pseudomyopia cannot be confused with the term “secondary myopia”, which includes transient myopic shifts caused by lenticular refractive index changes due to cataract [2,3], drugs [4] or diabetes mellitus [5], during and after hyperbaric oxygen therapy [6] or after blunt eye trauma with ciliar edema [7], or myopia associated with systemic syndromes [1,8]. The IMI defines secondary myopia as a myopic refractive state for which a single, specific cause can be identified that is not a recognized population risk factor for myopia development [1].

Pseudomyopia is the result of an increase in ocular refractive power due to an overstimulation of the eye’s accommodative mechanism [9,10,11,12]. The ciliary muscle, due either to continual overaction or other innervation effects, does not fully relax when objects at optical infinity are regarded [11]. Accommodation is not completely relaxed at optical infinity, and it is abolished by complete cycloplegia refraction [13]. Therefore, ocular refractive power is less myopic or more hypermetropic when ocular accommodation is relaxed. The difference between cycloplegic and non-cycloplegic refraction is one of pseudomyopia’s diagnostic signs. In fact, several authors define pseudomyopia as an apparent myopia that is acute in onset and disappears in the eyes when they are cyclopleged [9,10,11,14,15,16,17,18,19,20].

The aim of the current study was to review the literature for qualitative evidence about pseudomyopia as a condition where the increase in ocular refractive power is due to an overstimulation of the eyes, without convergence spasms and miosis.

## 2. Methods: Literature Search

A comprehensive literature search of PubMed and the Scopus database was carried out for articles in English published up to November 2021, without a data limit. The search terms and queries used were (pseudomyopia OR pseudo-myopia OR pseudo myopia). This review was reported following the preferred reporting items for systematic reviews and meta-analyses (PRISMA) guidelines [21]. A large number of them were excluded according the inclusion and exclusion criteria (Table 1). The titles and abstracts of all articles were reviewed, and articles requiring a full-text review were further identified. The reference lists of included studies were reviewed for additional articles.

## 3. Results

**Figure 1:**
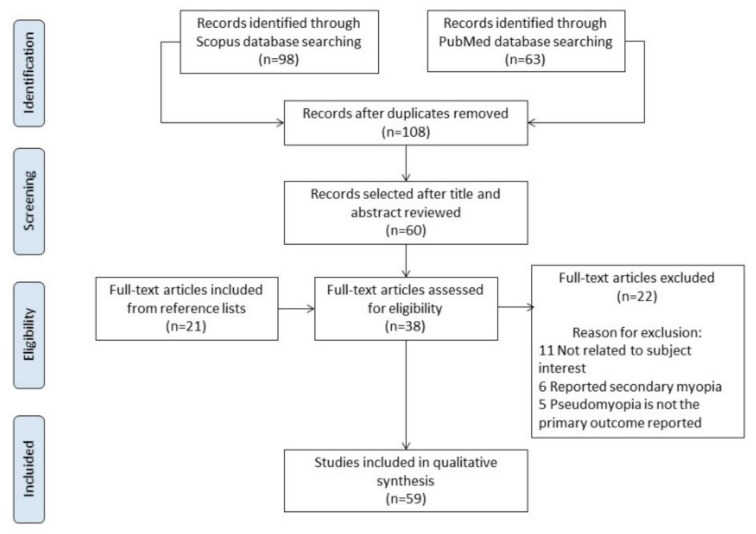
PRISMA flow diagram.

The initial search yielded 108 results after duplicates were removed. Following inclusion and exclusion criteria (Table 1), after the title and abstract were reviewed, 60 of them were selected. After the full text was revised, 22 were excluded; 11 were not related to the subject of interest, 6 reported secondary myopia and 5 reported pseudomyopia, but not as the primary outcome. A secondary search was carried out among the references included in the 38 selected. This process led to 21 studies being selected and revised for the present manuscript. Therefore, a total of 59 studies were included in the qualitative synthesis, including 21 case reports, 11 reviews without a meta-analysis, 6 guides and 21 prospective comparative cohort trials. The PRISMA [21] flow diagram is shown in Figure 1. 

### 3.1. Pathophysiology

Most of the studies referred to terms such as near work-induced transient myopia (NITM) [22,23,24] and accommodation spasms [10,14,19,25,26,27]. Immediately after accommodative efforts to focus on the near, the individuals with the condition have an inability to reduce the power of the crystalline lens rapidly and fully to focus afar. The magnitude of NITM reported ranged between 0.2 and 0.6 D. The decay time range reported was between 6.07 and 68.2 s, and can be induced following range times of 3 to 60 min of relatively close (a range of 0.12 to 0.25 m) near work. In addition, myopes are more susceptible to the near work after effect than hypermetropes [24] and emmetropes [28]. In fact, some authors reported pseudomyopia associated with excessive near work [12,22].

In addition, some authors suggest that NITM is one of the factors that contributes to the progression of myopia, with near work being one of the most important myopigenic environmental factors currently known [29,30,31,32]. In fact, Vasudevan and Ciuffreda showed that residual NITM may contribute to the progression of permanent myopia [33].

Walker published several case reports in 1946 that led him to define the terms pseudo-myopia and spasm of accommodation [10]. Before his publication, the author considered a spasm of accommodation to be a condition found in myopes, emmetropes and hypermetropes, in the young and in the middle-aged. However, he used the term pseudo-myopia only in young myopes. After the recompilation of several cases, he concluded that the term pseudo-myopia can be attributed to any refractive or age group. The confusion was of the etiological origin; in fact, pseudomyopia may be due to ciliary spasms. So, an accommodative spasm is a condition which occurs from excessive parasympathetic stimulation of the eye causing pseudomyopia due to ciliary muscle spasms [25]. In fact, other authors suggested exploring the possible impact of sympathetic innervation during intense near work [28]. In some cases, an accommodative spasm is associated with miosis and excessive convergence of the near reflex [17,34,35]. However, it may also exist as an isolated entity without convergence and miosis [12,14,22,25,36], which is in fact the target of the current review. 

In addition, some authors include instrument myopia [1] and night myopia [1,8,37] in the pseudomyopia category. In dim illumination during distance viewing, a small amount of accommodation may occur rather than be at a zero level. This is known as the dark focus of accommodation, and is responsible for the phenomenon known as night myopia [8,37]. However, Artal et al. showed myopic shifts lower than 0.50 D that only occurred at very low light conditions and after dark adaptation [37]. This may imply a limited practical impact in most subjects even if the situation is under fully natural conditions. 

Moreover, uncorrected hypermetropia or intermittent exotropia has been proposed for overstimulation of the eye’s accommodative mechanism. Patients with latent hyperopia who over-accommodate on refraction can yield a false myopic correction. This phenomenon is of interest in the refractive surgery sections. There are some case reports that show accommodative spasms after myopic photorefractive keratectomy [38] and laser-assisted in situ keratomileusis procedures [39]. 

Large exophoria or intermittent exotropia have been described as causes of pseudomyopia. Due to a deficiency in fusional convergence, such patients control their exodeviation with accommodative convergence resulting in pseudomyopia [15]. Sanker et al. reported the case of a 22-year-old, male myopic subject with intractable accommodative spasms that followed untreated intermittent exotropia, with no neurological abnormalities [26]. The authors theorized that the chronic state of accommodative spasms occurred as a result of a sustained over-accommodative response in an attempt to overcome a large angle exodeviation and maintain fusion. Furthermore, Jayakumar et al. published the case of a young 29-year-old healthy male with a diagnosis of basic exotropia, where blurred vision was not noted when uniocular visual acuity was measured [16]. The pseudomyopia was initially missed due to inadequate binocular vision testing, but this was only speculation, as the patient had initially presented blurred vision. The pseudomyopia persisted after strabismus surgery because of the residual angle, which he wanted to overcome by accommodative convergence. This required the use of cycloplegic agents to abolish the spasm.

There are other causes that generate pseudomyopia: emotional/psychological disorders [9,34,40,41,42,43], acquired brain injuries [18,25,36,40,44,45] and ocular traumas [22]. After brain injury, several structures associated with the control of accommodation can be injured. Kowal et al. published a retrospective analysis of ophthalmic manifestations in 164 patients with a head injury, and 19% presented pseudomyopia [44]. Post-traumatic pseudomyopia does not follow the course normally observed in functional cases [18]. In fact, London et al., 2003 [18] reported three post-traumatic pseudomyopia cases: one that improved in condition, another that did not improve completely and a third that did not improve at all. One of the reported cases was not pseudomyopia. After head trauma, the shift in the myopia evolution of the subject increased significantly, and the authors hypothesized that trauma influenced the ocular growth mechanism in some unknown manner [18]. Chan et al. published six new cases of post-traumatic pseudomyopia that did not manifest miosis or esotropia. In these cases, the complaint of blurred distance vision, which was readily rectified with glasses, was initially attributed to other neuro-ophthalmic consequences of head trauma [36]. In addition, McMurray et al. reported the case of a 28-year-old man with decreased visual acuity after sustaining closed head trauma in a motor vehicle accident 16 weeks earlier. The patient had a persistent accommodative spasm causing up to 5 D of pseudomyopia [45]. Bohlmann et al. also reported a patient with an accommodative spasm up to 9 years after head trauma, but with a lower grade of pseudomyopia, approximately 2.00 D [40]. Other authors reported one case associated with whiplash [25]. Several authors reported different evolutions and treatments because the mechanism of post-traumatic accommodative spasms is uncertain. Pseudomyopia after head trauma without signs of miosis or convergence spasms has been reported for several authors, and the majority were not resolved easily; in fact, some cases report dysfunction up to 9 years after the trauma [40].

### 3.2. Clinical Presentation

The most common symptoms of pseudomyopia are a blurred and variable distance vision and headaches [9,22,26,36]. There is a close correlation between unaided distance visual acuity and the amount of myopia; however, this correlation is not maintained in the presence of pseudomyopia. Fluctuations in distance visual acuity are due to fluctuations in accommodation, and can be observed as variations in not only visual acuity, but also in the retinoscopic reflex and, sometimes, changes in pupil diameter. Patients with an intermittent divergent squint complain of blurred vision when seeing binocularly, due to ciliary muscle contractions with accommodative convergence; however, this is not noted when uniocular visual acuity is measured. This condition can confuse pseudomyopia diagnosis [16]. On the other hand, the condition is bilateral, although there are some unilateral cases registered, as with those presented by Peinado et al. [14], Rutstein et al. [12], London et al. [18] and Hughes et al. [25], in which the condition was observed only when the binocular vision was disrupted. So, it is recommended to register monocular and binocular visual acuity.

The definitive sign of pseudomyopia is significantly more minus power in the manifest refraction than the cycloplegic refraction; this term has been named the gap refraction in the current review, and is registered in Table 2. This additional minus power cannot be eliminated with the standard refraction procedures used to relax accommodation at a distance, so ocular atropination is mandatory [27].

### 3.3. Assessment and Diagnosis

Based on the definition of pseudomyopia as a transient form of myopia due to overstimulation of the eye’s accommodative mechanism [9,10,11,12], the diagnosis of the condition can be confirmed when the non-cycloplegic refraction is more negative than the cycloplegic refraction. Table 2 summarizes this term and the main features of the reviewed case reports.

To achieve the manifest refraction, a careful subjective refraction based on retinoscopy results is recommended to determine the lowest minus lens power that achieves the best visual acuity [46]. In addition, cycloplegic refraction ensures an accommodation relaxation that allows the difference between both refraction values to be known. However, Demir et al. [47] found consistency between non-cycloplegic photoscreener measurements and cycloplegic autorefractometer measurements in patients with pseudomyopia due to accommodation spasms. 

The recommended dosage for cycloplegic refraction is 2 drops of 1% tropicamide or cyclopentolate given 5 min apart, and cycloplegic refraction should be performed 30 to 45 min after the first drop is instilled [48]. Some authors find both cycloplegic and standard clinical non-cycloplegic techniques acceptable, but others consider the cycloplegic autorefractomer technique as the gold standard [49]. However, the difference between non-cycloplegic and cycloplegic refraction (gap refraction) is different according to the baseline refractive status, and is a normal component of latent refractive error. Hypermetropes demonstrated a larger gap refraction than myopes [50,51,52], and in the normal myopic population without accommodative disorders, the gap refraction was <1.00 D. The *gap refraction* values reported by Mimouni M et al. and Sankaridurg P et al. were between 0.36 and 0.53 D [50] and 0.28 and 0.77 D [51], respectively. These values report a normal situation that some authors define as physiological pseudomyopia [13]. In fact, the *gap refraction* summarized in the current review shows values higher than 1.00 D that are not values of a physiological myopia, although there are some recorded under 1.00 D [18,36,38] (Table 2).

However, this is not the only clinical sign for detecting pseudomyopia. The presence of reduced and variable distance visual acuity, a low amplitude of accommodation for the patient’s age, more minus power in subjective refraction than in static retinoscopy, a leading accommodative response [18,26], fluctuations in retinoscopic findings and non-cycloplegic refractions or pupil reflexes could suggest a diagnosis of pseudomyopia, and indicate that a cycloplegic refraction should be performed. 

In addition, an increase in the fluctuations of the refractive power of an eye with negative spherical aberrations is another possible clinical sign [22,53]. Shetty et al. reported the role of aberrometry in a case report on an accommodative spasm, after myopic photorefractive keratectomy identified an internal defocus on aberrometry [38]. In this case it is interesting to observe that the *gap refraction* is not clinically relevant; however, the symptoms of the patients were resolved in cycloplegic conditions when the internal defocus was reduced. 

Ninomiya et al. also investigated changes in the spherical aberration of eyes with an accommodative spasm, presenting two case reports. Their results demonstrate that the excessive accommodative tone in eyes with an accommodative spasm is manifested objectively by negative spherical aberrations [22]. On the other hand, Artal et al. showed that spherical aberration does not play a significant role in night myopia [37]. 

Ocular examinations should include an assessment of extraocular movements and an orthoptic exam to identify convergence excess or spasms characterized by intermittent episodes of variable esotropia [9,14,17,34,54] or large exophoria [15,16,18,26]. A detailed orthoptic evaluation should be performed in all cases of an accommodative spasm before assuming that it is idiopathic [26].

It is important to discard organic processes, so some authors [12,16,19,35] recommend a neurological assessment with neuroimaging tests. In addition, the history for psychological triggers or stressors is important to consider [34] to investigate a possible neurotic/hysterical disposition [26,41,42].

In summary, it is important to carry out a detailed examination under cycloplegia because this is a key baseline data point for the diagnosis of pseudomyopia. In addition, some authors [14] reinforce this test to avoid errors in the diagnosis of slight myopic hypercorrections which frequently occur, and to avoid promoting accommodative spasms and myopia progression.

### 3.4. Treatment

The first step would consist of selecting management on the basis of etiology, if any is found [14]. The definitive treatment remains problematic because, in many cases, the etiology of the condition is unknown. However, the goal of treatment is to relax accommodation and eliminate pseudomyopia. When the etiology of a problem is not well understood, authors suggest looking for solutions in lower hierarchical branches. The most extreme management methods were reported by McMurray et al., involving the removal of the apparatus that is responding to the disrupted control of the neural input i.e., a clear lens extraction [45]. McMurray et al. reported the case of a 28-year-old man with decreased visual acuity after closed head trauma sustained in a motor vehicle accident 16 weeks earlier. Several structures thought to be associated with the control of accommodation were injured. The patient had a persistent accommodative spasm causing up to 7.0 D of pseudomyopia. As the patient’s pseudomyopia did not appear to resolve spontaneously and his rehabilitation was unable to progress because of the visual symptoms, it was decided to remove the lens and thereby stop the accommodative response, providing a stable baseline for a refractive correction [45].

The rest of the literature reviewed chooses less invasive treatment alternatives such as cycloplegic agents [14,16,17,18,22,25,26,36,38,39,40], plus lens additions for near work during cycloplegic treatment [14,17,18,20,36], prescriptions of manifest [18,25,36,40,55] or cycloplegic refraction for distance [36], base-in prisms [11,16,20,26,54,56] and vision therapies designed to relax accommodation [18,26] and improve fusional vergence ranges [17].

The most frequent pharmacological treatment is the utilization of cycloplegic drugs. This treatment option started with Bohlmann and France [40] in 1987, and is still in use today. Table 3 summarizes the historical evolution of the application of treatments, from the oldest to the most current. The inhibition of ciliary muscle contractions through muscarinic receptors endeavors to alter accommodative spasms. The most widely used drug is 1% cyclopentolate [16,17,22,38] and 1% atropine [14,17,18,25,40], but a defined regime has not been established. Some authors reported the use of 2% or 5% homatropine [18,26,36], and if, after a period of time, the drug was not effective in relaxing accommodation, the medication was switched to 0.25% scopolamine [18].

Due to the fact that there is a link between accommodation and convergence, the relaxation of convergence is used as a means of treatment for inducing the relaxation of accommodation [11]. This treatment may either take the form of orthoptics or base-in prisms [11,16,17,18,20,26,56]. In fact, this strategy was the first applied in patients, from 1928 [20] to 1956 [11] (Table 3). In 1928, Shaffer described a method to reduce accommodation and convergence to zero while the patient was viewing an object at near point. First, he placed a base-in prism to reduce the convergence to zero, and then the plus lens was added to reduce accommodative demand [20]. In 1930, Padman reported several cases in which the base-in prism was used to relax accommodation in an office training session and in the habitual glasses of the patients [56]. Other authors may even warrant the prescription of base-in prisms later if pseudomyopia recurs after cycloplegics are stopped [16].

Based on the premise that performing near-visual tasks for a prolonged time strains the ciliary muscle and may cause abnormalities in the accommodative function of a lens, Takada et al. investigated the visual-acuity-improving effect of a device utilizing far-stereoscopic videos [57,58]. The authors found significant increases in distance visual acuity in a group exposed to alternately repeating, negative and positive accommodation-viewing 3D videos, compared to the near-visual task group.

Accommodative training in post-traumatic pseudomyopia has been reported by some authors, but it is not clear if the normalization of the accommodative response was due to this type of exercise or the pseudomyopia was resolved spontaneously [18]. Other authors include accommodative and vergence training in the management of pseudomyopia associated with binocular disorders [17,26]. Shanker et al. applied accommodative training before surgical treatment to recovery motor fusion [26], and Laria et al. reported a case of pseudomyopia associated with a convergence spasm where visual training was applied after botulinum toxin treatment of the medial rectus [17].

Furthermore, near additions in bifocals to reduce the amount of accommodation have been used since 1928 [20] up to nowadays [14] (Table 3). Its application is shown in cases of esophoria associated with pseudomyopia [54], and Ciuffreda et al. suggested that in clinical practice, relatively high-powered near point lenses should be prescribed [23]. Nevertheless, they suggested revising the magnitude of the prescribed near-vision lens due to its effect on the near phoria via the AC/A ratio, as well as revising the patient’s compensatory near vergence ranges to preclude iatrogenically-induced blur, diplopia or more general asthenopia [23].

In the management of pseudomyopia, the revising authors combined the plus lens additions for near work during cycloplegic treatment, so the subjects restore their functional near vision [14,17,18,36].

An overcorrection of minus lenses is not a good strategy to resolve accommodative spasms; however, some authors reported the prescription of minus lens non-cycloplegic refractions as a supportive measure to reduce distance blur [18,25,36]. The prescription of cycloplegic refractions for distance has been reported in one case report, but the patients did not support the blurred vision and the strategy was changed to the prescription of a non-cycloplegic refraction [36].

Others authors had studied the effect of massage and acupuncture therapy in young pseudomyopia samples (5 and 16 years) during the 3 to 18 month period [59]. They concluded that combination therapy reported better effects in the treatment of pseudomyopia than massage therapy only. In fact, the published total effective rate of 92.2% in the treatment group (massage plus acupuncture) was greater than the 82.8% in the control group (only massage). However, during the treatment, the patients were encouraged to conduct more outdoor physical exercise, see more green plants and avoid poor eye-care habits. These recommendations were not monitories, so there could be bias in this study. Regarding neurotic/hysterical patients, minus lens may be prescribed initially for immediate relief [9], although emotional therapy is recommended. 

Attempts to break ciliary spasms through myotic drugs, cycloplegics with positive lenses or vision therapy, or by giving supportive measures such as prescribing minus lenses or multifocal intraocular lens implants in a refractory case, may be undertaken, but the clinician and patient should be aware of the guarded prognosis [18].

**Table 2:** Summary of the clinical features of the reviewed cases reports.

Author Year	Sex	Age	Symptoms/Signs	Diagnostic Test	Non-Cycloplegic Rx (D)	Cycloplegic Rx (D)	Gap Refraction (D)	Dx/Etiology	Treatment	After Treatment
Rutstein, Marsh-Tootle 2001 [12]	Female	27	Blurred distance vision in LE	VA Non-cycloplegic Rx Cycloplegic Rx Orthoptic exam Accomodative response Funduscopy Slit-lamp exam	RE plano LE −5.00	RE + 0.75 − 0.25 × 180 LE + 0.75 − 0.50 × 180	RE 0.75 LE 5.75	Pseudomyopia Unilateral LE accommodative spasm associated with excessive near work	Not treatment	Not reported
Ninomiya et al. 2003 [22]	Male	12	Blurred distance vision after a head/eye trauma soccer ball	VA Non-cycloplegic Rx Cycloplegic Rx Slit-lamp exam SA *	RE −2.00 LE −3.00 SA RE: −0.107 μm LE: −0.112 μm	RE plano LE plano	RE 2.00 LE 3.00	Pseudomyopia Accommodative spasm associated with eye trauma	Cyclopentolate 0.1%, 2 drops/day	Resolved SA RE: 0.020 μm LE: 0.031 μm
Ninomiya et al. 2003 [22]	Female	37	Blurred distance vision	VA Non-cycloplegic Rx Cycloplegic Rx Slit-lamp exam SA *	RE −11.25 LE −5.00 SA RE: −0.075 μm LE: −0.048 μm	RE −3.50 LE −3.00 SA RE: 0.027 μm LE: 0.022 μm	RE 7.75 LE 2.00	Pseudomyopia Accommodative spasm associated with excessive near work	Not reported	Not reported
Peinado et al. 2019 [14]	Female	10	Monocular decreased of vision	VA Non-cycloplegic Rx Cycloplegic Rx Orthoptic exam Papillary and macular OCT Neurophysiological studies	RE −6.00	RE +0.50	RE 6.50	Pseudomyopia Unilateral RE accommodative spasmIdiopathic	Atropine 1% daily (15 days) and +3D near-vision spectacles in RE	Resolved
Hughes et al. 2017 [25]	Female	34	Blurred distance vision in RE after a whiplash injury sustained	VA Non-cycloplegic Rx Cycloplegic Rx Orthoptic exam Funduscopy Slit-lamp exam Neuro-ophtalmology exam MRI	RE −3.50	Not reported		Pseudomyopia following a whiplash type injury	Far vision minus lens Atropine 1%	Not resolved
Laria et al. 2014 [17]	Female	8	Headaches and blurred vision	VA Non-cycloplegic Rx Cycloplegic Rx Orthoptic exam Funduscopy Slit-lamp exam Neuro-ophtalmology exam	RE −9.75 − 0.25 × 65°LE −7.75 − 0.75 × 65°	RE +0.75 LE +0.75 D	RE 10.50 LE 8.50	Pseudomyopia Esotropia, spasm of the near reflex	Cyclopentolate 1.0%, 2 drops/day Atropine 1%, 1 drop/day and near-vision glasses. Botulinum toxin in the medial rectus Visual therapy	Resolved
Shetty et al. 2015 [38]	Female	33	Blurred distance vision and headache after one month PRK for myopia	VA Non-cycloplegic Rx Cycloplegic Rx Slit-lamp exam Aberrometry †	RE −0.75 − 0.50 × 165° LE −0.50 × 20° Internal defocus RE: 1.019 μm LE: 0.366 μm	RE −0.50 × 165 LE −0.50 × 20° Internal defocus RE:0.142 μm (86% decreassed) LE:0.230 μm(36% decreased)	RE −0.75 LE plano	Pseudomyopia Accommodative spasm	Cyclopentolate 1%, 3 drops/day	Resolved
Airiani S, Braunstein RE 2006 [39]	Female	41	Severe headache after undergoing LASIK surgery.	VA Non-cycloplegic Rx Cycloplegic Rx Slit-lamp exam Funduscopy	RE −2.25 − 0.50 × 170° LE plano	RE plano LE +0.75	RE 2.25 LE 0.75	Pseudomyopia	Cyclopentolate 1%	Patient lost to follow-up
Shanker et al. 2012 [26]	Male	22	Headaches and blurred vision	VA Non-cycloplegic Rx Cycloplegic Rx Orthoptic examMEM Funduscopy	RE −10.00 D LE −10.00 D	RE −2.25 LE −1.50	RE 7.75 LE 8.50	Pseudomyopia Accommodative spasm	Homatropine 2%, 2 drops/day for 10 days Accommodative training	AS resolved, but after exotropia intermittent
Jayakumar et al. 2012 [16]	Male	29	Squint and blurred vision	VA Cycloplegic Rx Orthoptic exam	Not reported	RE plano LE plano		Basic exotropia	Bilateral lateral rectus recession	Blurred vision after strabismus surgery
			Blurred vision after strabismus surgery	VA Non-cycloplegic Rx Cycloplegic Rx Orthoptic exam NRA	RE −2.00 − 0.50 × 90° LE −2.75	RE plano LE plano	RE 2.25 LE 2.75	Pseudomyopia	Cyclopentolate 1%, 3 drops/day Prisms	Not reported
Bohlmann BJ, France TD 1987 [40]	Female	19	Blurred distance vision after trauma	VA Non-cycloplegic Rx Cycloplegic Rx Orthoptic exam Neuro-ophtalmology exam MRI	RE −1.50 LE −1.50	RE 0.25 LE 0.25	RE 1.75 LE 1.75	Pseudomyopia after a closed head trauma	Atropine 1% and bifocals	Resolved after 9 years
London et al. 2003 [18]	Female	15	Blurred distance vision after closed head trauma	VA Non-cycloplegic Rx Cycloplegic Rx Orthoptic exam MEM Neurophysiological studies	RE −1.50 LE−1.50 − 0.50 × 175°	RE +0.50 LE + 0.25 − 0.25 × 180°	RE 2.00 LE 1.75	Pseudomyopia Accommodative spasm associated with head trauma	Accommodative rock exercises	Resolved
London et al. 2003 [18]	Male	25	Blurred distance vision for a year.	VA Non-cycloplegic Rx Cycloplegic Rx Orthoptic exam MEM Neurophysiological studies	RE −2.75 LE −2.50	RE −0.50 LE −0.25	RE 2.25 LE 2.25	Pseudomyopia Accommodative spasm associated with Parinaud’s syndrome Exotropia	Accommodative rock exercises Atropine and near-vision spectacles Far vision minus lenses Strabismus surgery	Partially resolved Eventually he required far vision minus lenses
London et al. 2003 [18]	Female	36	Blurred and variable distance vision after closed head trauma.Pupillary asymmetry	VA Non-cycloplegic Rx Cycloplegic Rx Orthoptic exam Neuro-ophtalmology exam Funduscopy Slit-lamp exam	RE −1.50 − 1.00 × 175° LE −1.75 − 1.25 × 157°	RE −0.25 LE −0.25	RE 1.25 LE 1.50	Pseudomyopia Accommodative spasm associated with head trauma	Homatropine 5% Scopolamine 0.25% Bifocal glasses +2.00 D	Partially resolved She required pharmacologic drops and bifocal glasses
London et al. 2003 [18]	Male	17	Blurred distance vision without correction and blurred near vision with correction unilateral for a year after head trauma	VA Non-cycloplegic Rx Cycloplegic Rx Orthoptic exam Accommodative response (MEM) and amplitudes	LE −2.25	LE −0.25	LE 2.00	Pseudomyopia Unilateral LE accommodative spasm associated with head trauma	Bifocal glasses RE −0.50 Ad +0.75 LE −2.00 Ad +1.75	Partially resolved He required bifocal glasses
Chan RV, Trobe JD 2002 [36]	Male	30	Blurred distance vision after trauma	VA Non-cycloplegic Rx Cycloplegic Rx Orthoptic exam Neuro-ophtalmology exam MRI	RE −1.50 LE −1.50	RE plano LE plano	RE 1.50 LE 1.50	Pseudomyopia after a closed head trauma	Manifest Rx	Not resolved
	Male	20			RE −5.00 LE −5.00	RE −3.25 LE −3.25	RE 1.75 LE 1.75		Manifest Rx	Not resolved
	Male	18			RE −1.50 − 0.75 × 93° LE −2.50 − 0.50 × 96°	RE +0.75 − 1.50 × 90° LE +0.25 − 1.25 × 70°	RE 1.50 LE 2.75		Cycloplegic Rx Manifest Rx	Not resolved
	Male	17			RE −2.50 − 0.50 × 10° LE −2.50	RE −1.00 − 0.25 × 10° LE−0.75 − 0.25 × 150°	RE 1.50 LE 1.75		Homatropine and bifocals	Not resolved
	Male	16			RE −2.00 LE −1.25 − 0.75 × 55°	RE −1.25 LE −0.50 − 0.75 × 55°	RE 0.75 LE 0.75		Manifest Rx	Not resolved
Mc Murray et al. 2004 [45]	Male	28	Decreased VA after closed head trauma sustained in a motor vehicle accident 16 weeks earlier	VA Non-cycloplegic Rx Cycloplegic Rx Orthoptic exam Axial length Computerized tomography	NR	NR	RE 4.00 LE 5.25	Pseudomyopia Accommodative spasm associated with head trauma	Unsatisfactory despite a variety of cycloplegic and refractive corrections Finally, sequential clear lens extraction was selected.	Resolved VA was N5 with +2.50 D reading glasses
Park et al. 2021 [55]	Female	33	Blurred distance vision after	VA Non-cycloplegic Rx Cycloplegic Rx Orthoptic exam Accomodative response Funduscopy Slit-lamp exam Biometry	RE −2.34 (SE) LE −2.50 (SE)	RE −0.26 (SE) LE 0.13 (SE)	RE 2.08 LE 2.63	Pseudomyopia with paradoxical accommodation	Glasses −1.00 D	She is in monitorization
Nguyen et al. 2020 [43]	Female	10	Painless vision loss in both eyes	VA Non-cycloplegic Rx Cycloplegic Rx Orthoptic exam Accomodative response Funduscopy Slit-lamp exam Biometry	RE −8.50 − 0.50 × 57° LE −9.25 − 0.50 × 153°	RE +0.75 − 0.50 × 7° LE + 0.75 − 0.50 × 47°	RE 9.25 LE 10.00	Accommodative spasm associated with conversion disorder	Atropine 0.5%–0.1%–0.01% in both eyes once daily-10 weeks	Atropine drops discontinued

D: diopter; Dx: diagnosis; SE: spherical equivalent; NR: not reported; VA: visual acuity; RE: right eye; LE: left eye; Gap refraction: difference between cycloplegic and non-cycloplegic refraction; OCT: optical coherence tomography; MRI: magnetic resonance imaging; NRA: negative relative accommodation; SA: Spherical aberration; MEM: Monocular Estimation Method to measure accommodative response. * Spherical aberration measured by Hartmann-Shack wavefront aberrometer in central 4-mm zone. † Aberrometry measured by iTrace Visual Function Analyzer; Tracey Technologies.

**Table 3:** Historical evolution of treatments.

Author	Year	Cycloplegic Agents	Plus Lenses	Manifest Rx	Prism Base in	Orthoptics
Shaffer [20]	1928		x		x	
Padman [56]	1930				x	
Hathaway [54]	1930				x	
Willians [11]	1956				x	
Bohlmann BJ, France TD [40]	1987	x		x		
Ciufreda [23]	1999					
Chan RV, Trobe JD [36]	2002	x	x	x		
Ninomiya et al. [22]	2003	x				
London et al. [18]	2003	x	x	x		x
Airiani S, Braunstein RE [39]	2006	x				
Shanker et al. [26]	2012	x				x
Jayakumar et al. [16]	2012	x			x	
Laria et al. [17]	2014	x	x			x
Shetty et al. [38]	2015	x				
Hughes et al. [25]	2017	x		x		
Peinado et al. [14]	2019	x	x			
Nguyen et al. [43]	2020	x				
Park et al. [55]	2021			x		

## 4. Conclusions

There are various terms to define the condition that may lead an increase in ocular refractive power due to overstimulation of the eye’s accommodative mechanism, and the term accommodative spasm is the most cited. However, in several cases the accommodative spasm is associated with miosis and excessive convergence as part of the near reflex, where pseudomyopia is not the main clinical sign. Therefore, it is necessary to differentiate an accommodative spasm with pseudomyopia, and define it as an isolated entity in which convergence and miosis are secondary clinical manifestations, so that the terms pseudomyopia and accommodative spasm are used interchangeably. The authors of the current review suggest using the terms convergence spasm or near reflex spasm to reference cases where pseudomyopia is not the primary sign.

The most common symptom of pseudomyopia identified in the review has been blurred and variable vision. On the other hand, the majority of the reviewed authors identify the significantly greater minus power in the non-cycloplegic refraction than the cycloplegic refraction (gap refraction in the current review) as a definitive sign of pseudomyopia. In consequence, the ocular refractive under atropination is mandatory. However, there are others assessments to clarify the diagnosis; the spherical aberration evaluation is one of these, but there are few studies about it. 

Due to the link between accommodation and convergence, the literature review includes a complete orthoptic evaluation and neurological assessment with neuroimaging tests to discard organic processes. Generally, there is uniformity in the assessment and diagnosis of the condition; however, there is no consensus on management, and the literature describes a range of treatments. Definitive treatment remains problematic because, in many cases, the etiology of the condition is unknown. The literature reviewed chose treatment alternatives such as cycloplegic agents, plus lens additions for near work during cycloplegic treatment, prescriptions of manifest or cycloplegic refraction for distance, base-in prisms and vision therapies designed to relax accommodation and improve fusional vergence ranges. The common goal of treatment is to relax accommodation and thus eliminate pseudomyopia, but the strategies to achieve this are different.

This review has warned that there is agreement on the assessment and diagnosis of the condition; however, there is no consensus on management, and the literature describes a range of treatments.

## Figures and Tables

**Table 1:** Inclusion and exclusion criteria.

Inclusion Criteria
Pseudomyopia caused by an increase in ocular refractive power due to overstimulation of the eye’s accommodative mechanism.
Explicit mention of pseudomyopia as the primary outcome reported.
**Exclusion Criteria**
Not related to subject interest.
Reported secondary myopia.
Reported pseudomyopia, but it is not the primary outcome.

## Data Availability

Not applicable.
